# Eucalyptus saponin- and sophorolipid-mediated desorption of polycyclic aromatic hydrocarbons from contaminated soil and sediment

**DOI:** 10.1007/s11356-022-23562-z

**Published:** 2022-10-22

**Authors:** Thiloka Kariyawasam, Paul D. Prenzler, Julia A. Howitt, Gregory S. Doran

**Affiliations:** 1 School of Agricultural, Environmental and Veterinary Sciences, Charles Sturt University, Wagga Wagga, NSW 2678 Australia; 2 Gulbali Institute, Charles Sturt University, Wagga Wagga, NSW 2678 Australia

**Keywords:** PAHs, Soil, Sediment, Desorption, Sophorolipid, Eucalyptus saponin, Stability, Toxicity

## Abstract

The potential for biosurfactant-mediated desorption of polyaromatic hydrocarbons (PAHs) was evaluated using PAH-spiked soil and sediment. PAH desorption behaviors and toxicity of novel saponin biosurfactant extracted from *Eucalyptus camaldulensis* leaves and sophoro-lipid biosurfactant were investigated. Their PAH desorption efficiencies were compared with rhamnolipid biosurfactant and the industrial-chemical surfactant, Tween 20. Based on the emulsification indices, the salt tolerance of surfactants up to 30 g/L NaCl followed the order of saponin > Tween 20 > sophorolipid > rhamnolipid, while the thermal stability over the range of 15 to 50 °C was in the order of sophorolipid > rhamnolipid > saponin > Tween 20. The saponin biosurfactant emulsion demonstrated the highest stability under a wide range of acidic to basic pHs. PAH extraction percentages of saponin and sophorolipid under the optimized surfactant concentration, volume, and incubation time were 30–50% and 30–70%, respectively. PAH desorption capacities of saponin and sophorolipid were comparable to that of rhamnolipid and Tween 20 for all matrices. Sophorolipid more efficiently desorbed low molecular weight PAHs in soil and sediment compared to the other three surfactants. Microbial respiration was used to determine biosurfactant toxicity to the soil/sediment microbiome and indicated no inhibition of respiration during 60 days of incubation, suggesting that sophorolipid- and saponin-mediated remediation may be sustainable approaches to remove PAHs from contaminated soils and sediments.

**Supplementary Information:**

The online version contains supplementary material available at 10.1007/s11356-022-23562-z.

## Introduction

Polyaromatic hydrocarbons (PAHs) are toxic environmental pollutants that resist environmental degradation due to their highly hydrophobic nature and, as such, can accumulate in soil, sediments, air, aquatic organisms, and plants. Soils and sediments are considered the primary reservoir for PAH accumulation, and concerns over their adverse health effects have resulted in many studies on the remediation of contaminated soil. Although several different techniques exist, bioremediation is showing particular promise as a safe, sustainable, and cost-effective option. Consequently, the degradation of PAHs by microorganisms has gained more attention in recent studies. However, the low aqueous solubility of PAHs reduces the efficiency of bioremediation because PAHs remain tightly bound to soil and are not readily bioavailable for microbial degradation. Incorporation of foreign microbes into contaminated sites (bioaugmentation) to enhance PAH degradation will still not overcome this problem, and, as a result, improved PAH desorption from soil to enhance their bioavailability is the main obstacle to be overcome to improve the viability of bioremediation. The application biosurfactants produced by non-pathogenic microbes to soil may solve the issue of desorption because they are biologically active compounds produced by microorganisms and plants, which have surface-active properties and produce micelles (Li and Chen 2009).They have the potential to improve the solubility of hydrophobic PAHs, while offering advantages such as lower toxicity, higher biodegradability, and effectiveness under a wide range of extreme environments compared to chemical surfactants (Lee et al. 2018).The application of biosurfactants to the soil where PAH microbial degradation takes place may allow enhanced remediation of contaminated soil in situ. When the concentration of biosurfactant is greater than the critical micelle concentration (CMC), biosurfactant monomers associate to form micelles, bilayers, and vesicles, enabling biosurfactants to reduce both surface tension and the interfacial tension (Jahan et al. 2020), which can enhance the solubility of nonpolar organic compounds by forming stable emulsions. Hydrocarbons are dissolved in the hydrophobic cores of the micelles produced by surfactant, and these complexes can be directly absorbed by microbes or diffuse into the pore water to make them available for microbial uptake (Li and Chen 2009).

Among bacterial species, *Pseudomonas aeruginosa* is one of the prominent candidates involved in biosurfactant production (Patowary et al. 2015), but, due to its pathogenicity, it is vital to investigate non-pathogenic species capable of biosurfactant production to improve safety. Cost of production must also be a consideration for the industrial production of biosurfactants, which can limit the application of surfactants such as rhamnolipid biosurfactants from *Pseudomonas aeruginosa*, which has a high production cost. Freitas et al. (2016) described the utilization of sophorolipid biosurfactants produced by the yeast *Candida bombicola* as a dispersant of petroleum and by-products spilled in oceans. The sophorolipid biosurfactant produced from *Candida* yeast is a non-purified product from non-toxic biodegradable industrial waste and is considered a promising alternative for the remediation of petroleum products. Compared to bacterial biosurfactants, sophorolipids are mainly derived from non-pathogenic yeast species (Freitas et al. 2016) and can be produced in larger amounts at a low cost by fermenting feedstock, such as animal fat, deproteinized cheese whey, soy molasses, restaurant waste oil. However, the application of sophorolipids as biosurfactants in PAH bioremediation in soil is so far limited to phenanthrene (Schippers et al. 2000), and its potential to remediate soil contaminated with a mixture of small and large PAHs is yet to be explored.

In addition to microbially produced surfactants, some plants are also known to produce biosurfactants. Blyth et al. (2015) used surfactants from the leaf extract of the Australian red ash tree in effective remediation of PAH-contaminated soil. Eucalyptus leaves also can be considered a reservoir of biosurfactants, which may be associated with the activity of microbes resident in leaves or produced as secondary metabolites in the plant (Aulwar and Rs 2016; Kuppusamy et al. 2016). Although Kuppusamy et al. (2016) isolated the PAH-degrading and surfactant-producing bacteria from koala feces, it is unclear whether the surfactants are produced by microbes residing in the koala’s gut or in eucalyptus leaves prior to ingestion. Hence, further investigation of the effectiveness of PAH desorption by the biosurfactants produced in eucalyptus leaves is warranted due to the lack of research in this area. The present study aims to investigate ways in which PAH desorption from soil can be improved to allow greater bioavailability of PAHs to soil microbes to enhance PAH degradation and improve the rate of bioremediation of the soil. To do this, the effectiveness of sophorolipid biosurfactant and the biosurfactant extracted from leaves of *Eucalyptus camaldulensis* on desorption of PAHs from soil and sediment will be studied. Furthermore, efficiency of the above biosurfactants will be compared with the widely used rhamnolipid biosurfactant and the Tween 20 industrial-chemical surfactant. The toxic effect of surfactant amendment on soil/sediment microbial communities was also studied by considering microbial respiration.

## Materials and methods

### Chemicals and plant materials

The PAH standards phenanthrene (PHE), pyrene (PY), chrysene (CHY), and benzo[a]pyrene (BAPY) and their deuterated analogs (phenanthrene-d10, pyrene-d10, chrysene-d10, and benzo[a]pyrene-d12) were purchased from Sigma-Aldrich, USA. HPLC-grade acetonitrile and acetone were purchased from Chem-Supply (RCI labscan, Australia).The rhamnolipid biosurfactant (90% purity) extracted from *Pseudomonas aeruginosa* was purchased from Sigma-Aldrich, Australia. The sophorolipid biosurfactant (> 80% purity) from the yeast *Candida bombicola* produced by Cayman Chemical, USA, was purchased from Sapphire Biosciences, Australia. Industrial-chemical surfactant Tween 20was purchased from Sigma-Aldrich, USA. Leaves of *Eucalyptus camaldulensis* were used to extract saponin biosurfactants. Standard saponin was purchased from Alfa Aesar (Thermo Fisher Scientific, Australia).

### Soil and sediment for PAH spiking studies

Soil was collected from an agricultural field in Yanco, NSW, Australia (34°360ʹ S, 146°240ʹ E) and was characterized as a brown chromosol. Sediment was collected from Bullenbung creek, Galore (35°182ʹ S, 146°944ʹ E), NSW, Australia. Characteristics of the soil and sediment have been previously reported (Kariyawasam et al. 2022). Soil and sediment used for spiking were sterilized by gamma irradiation (Steritec, NSW, Australia) to minimize microbial activity.

### Spiking of soil and sediment

Spiking of the soil and sediment was carried out as described by Kariyawasam et al. (2022). Two grams of blank soil or sediment was weighed into glass screw cap scintillation vials (20 mL) in triplicate and covered with acetone (1800 µL). The solutions were spiked with PAHs (800 µL, 5 μg/mL), and the acetone was evaporated under a gentle stream of nitrogen, resulting in PAH concentration in soil and sediment of 2 mg/kg. For the full method validation, 400 µL was spiked from 2.5, 5, 10, 20, and 50 μg/mL PAH solutions onto soil/sediment to obtain 0.5, 1, 2, 4, and 10 mg/kg, respectively, as the final PAH concentrations.

### Extraction and characterization of the saponin biosurfactant

Eucalyptus leaves were oven dried at 40 °C for 4 days and crushed into pieces. The extraction of saponin was carried out as described by Hajimohammadi et al. (2016). Dried and crushed eucalyptus leaves (10.0 g, *n* = 3) were defatted with hexane (100 mL) for 48 h using a Soxhlet apparatus. Defatted samples were then extracted with methanol for 24 h and concentrated under a vacuum. The crude extract was suspended in deionized water and partitioned with n-butanol. Next, the aqueous fraction was discarded, and diethyl ether was gradually added to the n-butanol partition to precipitate the saponin fraction. The solution was cold centrifuged (10 °C) at 3041 × g for 5 min, and the solid precipitate was recovered. The precipitate was frozen at − 80 °C for 24 h and then freeze dried, providing a percentage yield of 1.04 ± 0.10% for the crude saponin biosurfactant. The extracted saponin was characterized using Fourier transform infrared (FT-IR) spectroscopy and compared with a standard saponin. The FT-IR spectrum was obtained using a Spectrum Two™ FT-IR spectrometer (PerkinElmer, USA).

### Determination of the CMC of the biosurfactants

Aqueous solutions of surfactants in the range of 10–100 mg/L were prepared, and the surface tension was measured using a digital tensiometer at 20 °C (ANALITE Model 2141, McVan Instruments, Australia) according to the Wilhelmy plate method. CMC was determined by the intersection between the straight lines formed in the surface tension graph as a function of surfactant concentration.

### Emulsifying activity of the biosurfactants and the stability studies

Emulsification activities of biosurfactants (100 mg/L) were determined by measuring the emulsification index after 24 h (*E*_24_). Gasoline (2 mL), from a fuel station, was added to the same amount of the surfactant solution (*n* = 3) and vortexed for 2 min. Then, the solutions were allowed to settle down for 24 h at room temperature (20 °C) and the *E*_24_ values were calculated using the following equation (Hajimohammadi et al. 2016):

1 $$E_{24}=\frac{\mathrm{Height}\;\mathrm{of}\;\mathrm{the}\;\mathrm{emulsion}\;\mathrm{layer}}{\mathrm{Total}\;\mathrm{height}\;\mathrm{of}\;\mathrm{the}\;\mathrm{solution}}\times100$$

The effect of various environmental factors on the stability of surfactant emulsions was determined by studying the effects of temperature (15 °C, 20 °C, and 50 °C by incubation in temperature-controlled water baths for 24 h), pH (2, 7, 10, and 12 with 1 M HCl and 1 M NaOH), and salinity (varying amounts of NaCl 3, 10, 30 g/L) on the emulsification indices of the surfactants.

### Biosurfactant-aided desorption of PAH in contaminated soil and sediment

#### Experimental design to obtain the optimum conditions

A second-order multivariate Box–Behnken experimental design (BBD) was employed to identify optimal experimental conditions through a response surface study. Three experimental parameters—concentration (*X*_1_), surfactant volume (*X*_2_), and incubation time (*X*_3_)—were selected as independent variables. Percentage desorption was the response for the combination of the independent variables. The three levels of each variable were designated as − 1, 0, and + 1 and represent low, medium, and high values, and are listed in Table 1. Based on a factorial design, a total of 30 experiments were performed, including six center points as described in Table S1 in the supplementary information.

**Table 1:** Selected factors and levels for surfactant-mediated desorption of PAHs according to the BBD

Factor	Code	Level		
		− 1	0	+ 1
Concentration (mg/L)	*X*_1_	0.5 × CMC	1.25 × CMC	2 × CMC
Surfactant volume (mL)	*X*_2_	5	10	15
Incubation time (days)	*X*_3_	3	5	7

The mathematical relationship of the response for *X*_1_, *X*_2_, and *X*_3_ can be approximated by the second-order polynomial equation.

2 $$Y=\beta_0+\beta_1X_1+\beta_2X_2+\beta_3X_3+\beta_{12}X_1X_2+\beta_{13}X_1X_2+\beta_{13}X_1X_3+\beta_{23}X_2X_3+\beta_{11}X_1^2+\beta_{22}X_{2}^{2} + \beta_{33}X_3^3$$

where *Y* represents the response variable, *ß*_0_ is the intercept of the model, *ß*_1_, *ß*_2_, *ß*_3_, and *ß*_12_, *ß*_13_, *ß*_23_ are linear and interaction coefficients, respectively (Dahaghin et al. 2017). *ß*_11_, *ß*_22_, *ß*_33_ are quadratic coefficients. The analysis of the experimental design and optimization was performed using Minitab 17 and Sigma Plot 13 software. The optimal extraction conditions were established through regression analysis and response surface plots.

#### Desorption of PAHs to surfactant solutions

PAH-spiked sterilized soil and sediment (2 g) were mixed with 5,10,15 mL of the surfactant solutions (0.5 × CMC, 1.25 × CMC, 2 × CMC). The soil slurry samples (*n* = 3) were mechanically mixed by inversion for 3–7 days and centrifuged. The supernatants were spiked with the internal standards (50µL, 10 ppm) to account for the losses during the filtration and evaporation steps and minor variations in resuspension volume. Supernatants were then mixed with the same volume of hexane and vortexed for 4 min and allowed to settle for 1 h. The hexane layer was evaporated under a gentle stream of nitrogen and reconstituted with 2 mL of hexane:acetone (1:1). Filtered (0.22-µm PTFE) extract was analyzed by GC–MS. Under the most favorable experimental conditions, recovery efficiencies (in the range of 0.5–10 mg/kg) were determined to further validate the proposed method. Statistical analysis was conducted using one-way ANOVA and Tukey’s post hoc test (Wawra et al. 2018). A two‑tailed *P* < 0.05 was considered statistically significant.

#### GC–MS analysis of PAHs

PAHs were determined using gas chromatography with a mass selective detector (Agilent 8890A GC coupled to a 5977B MSD, 7693A). The PAHs were separated using a 30-m high-resolution glass capillary column DB5-MS (i.d., 0.25 mm; 0.25-µm film) (J&W Scientific, Agilent Technologies, DE, USA). The oven temperature was held initially at 90 °C for 1 min, and then ramped to 200 °C at 20 °C/min. The temperature then increased to 210 °C at 3 °C/min and to 290 °C at 5 °C/min followed by a 10-min hold. The helium column flow rate was 1.7 mL/min with a source temperature of 300 °C.

#### Search for oxy-PAHs in mass spectra

The possibility of generating oxy-PAHs during the surfactant treatments was studied by searching the NIST library for mass spectra of oxy-PAHs that matched the mass spectra of peaks in the GC–MS chromatograms. Seventeen commonly found oxy-PAHs were considered (Staffan et al. 2007). All identifications are tentative, as no authentic standards were used for comparison.

### Mass transport kinetics

The experiments were conducted using the above-mentioned method using the optimum soil-to-surfactant ratio and surfactant concentration, and the extent of PAH desorption with time was studied by sampling at days 1, 2, 3, 4, and 5.

### Application of biosurfactants to aged soil and sediment and naturally incurred soil

Sterilized soil and sediment were spiked with PAHs (2 mg/kg) in triplicate before incubation for 30 days at room temperature. Soil naturally contaminated by petrochemicals was collected near a diesel pump at Wagga Wagga, NSW (35°03′09.4ʺS 147°21′02.7ʺE) (incurred soil). Surfactants were applied to air dried, the naturally incurred soil and the aged, spiked soils (*n* = 3) under the optimized conditions previously identified and analyzed by GC–MS. PAHs in the incurred soil were extracted by eucalyptus oil extraction and quantified (Kariyawasam et al. 2022)to obtain the initial PAH concentrations in the soil. Percentage desorption of PAHs in incurred soil was determined relative to the amounts of PAHs quantified.

### Microbial respiration in surfactant-amended soil and sediment

Soil microbial respiration was measured in non-sterile PAH-spiked (2 mg/kg) and non-spiked soil and sediment samples (20 g) in the presence and absence of surfactants (500 mg/L) using the method described by Bartha and Pramer (1965) . Surfactant solutions (1000 mg/L) prepared in deionized water were sprayed into soil/sediment samples in 1-L glass jars (*n* = 4) using a hand sprayer to achieve 70% field capacity. A Petri dish containing 10 mL of 0.5 M NaOH solution was placed inside the jar containing soil/sediment to trap the evolved CO_2_. Jars containing soil/sediment with no surfactant added were used as controls, and jars with no soil/sediment were used as the blanks. Sealed jars were aerobically incubated at 22 °C for 60 days. NaOH solutions were taken out after 1, 7, 14, 21, 28, 35, 42, 49, and 60 days of incubation, and a fresh NaOH solution was placed into each jar. BaCl_2_ (1 M, 1 mL) was added to the NaOH solutions, which were titrated with 0.5 M HCl using 0.1% phenolphthalein as the indicator. The CO_2_ evolved was calculated from the difference in normality between NaOH blanks and samples.

## Results and discussion

### Extraction and characteristics of saponin biosurfactant

**Fig. 1:**
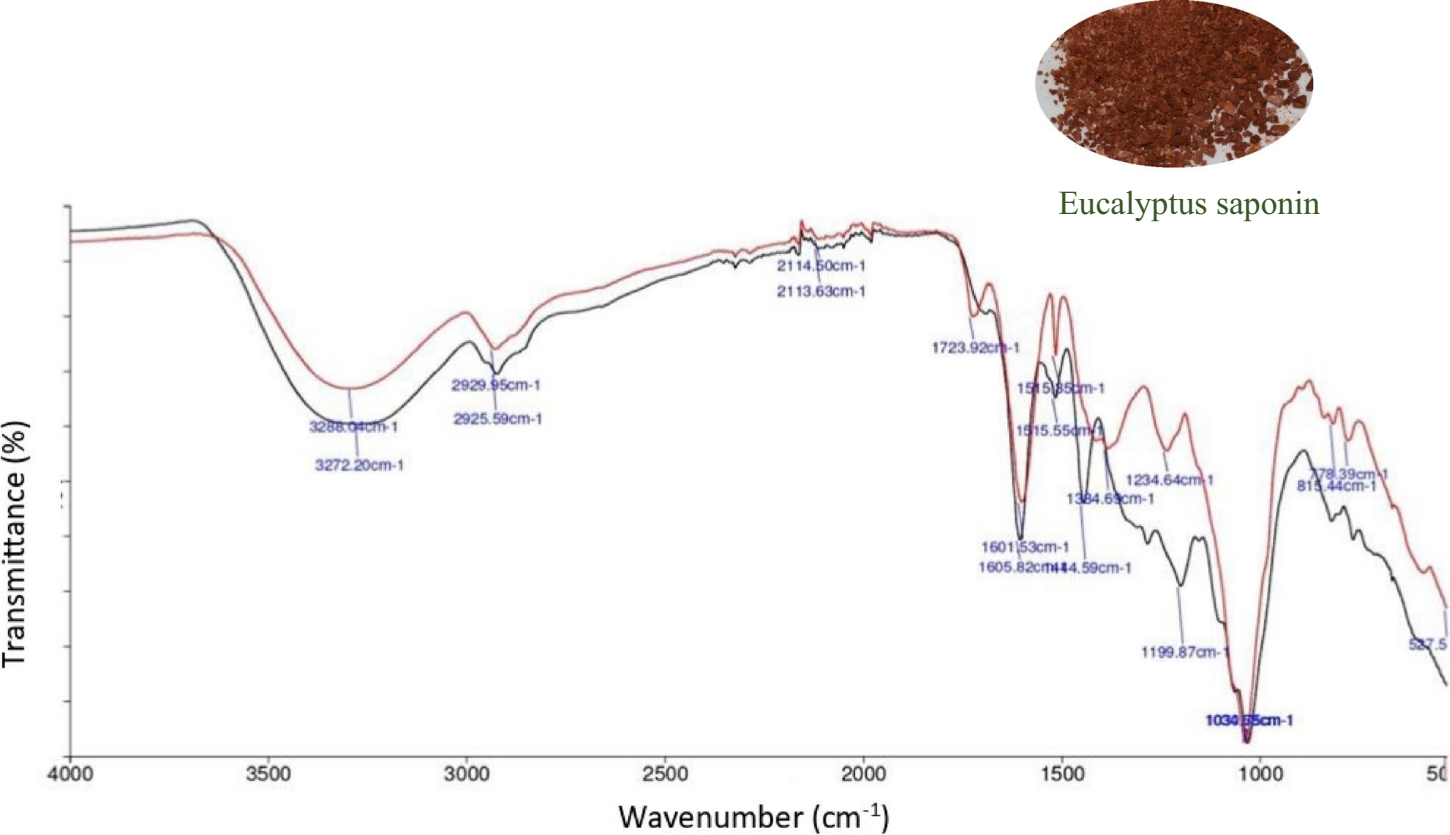
FT-IR spectra of the extracted saponin biosurfactant (  ) and the standard saponin (  )

According to the FTIR spectrum of the extracted saponin (Fig. 1), the broad absorbance at 3272 cm^−1^ corresponds to hydroxyl groups (O–H). The band at 2926 cm^−1^ could be related to C–H symmetric stretching. A band at 1606 cm^−1^ may be assigned to a C = C stretch, and C = C bending vibration may be evinced by the peak at 821 cm^−1^. In-plane bending vibration of C–H was found to be at 1445 cm^−1^. The peaks at 1030 cm^−1^ and 1199 cm^−1^ may be relevant to the stretching vibrations of C–O. Characteristics of the IR spectrum for the extracted saponin were in accordance with the IR spectrum for the standard saponin, as presented in Fig. 1. Further, similar IR spectra were reported for the saponins extracted in previous studies (Hajimohammadi et al. 2016; Yu and He 2018). As this is the first report of the FT-IR spectrum for eucalyptus saponin, further analysis and purification steps are necessary to provide an exact classification and structure elucidation of the extracted saponin.

### Comparison of the CMCs of the surfactants

Surfactants spontaneously form micelles in aqueous solutions at concentrations above the CMC due to hydrogen bonding, hydrophobic, and van der Waals interactions (Jahan et al. 2020). Solutions below their CMC are monomeric and dispersed, while above their CMC they are self-aggregated to form micelles, which are thermodynamically stable nanostructures.As the surfactants facilitate mass transfer by mobilization of PAHs at the concentrations below CMC and solubilization above the CMC (Bezza and Nkhalambayausi-Chirwa 2015), knowing the CMCs of the surfactants is important when conducting surfactant studies. As exhibited in Fig. S1, CMCs of the studied biosurfactants were in the order of sophorolipid (50 mg/L) > rhamnolipid (40 mg/L) > saponin (30 mg/L). In this study, Tween 20 was selected as a representative synthetic surfactant to compare with the performances of biosurfactants. Based on the CMC values, Tween 20 showed the highest CMC (60 mg/L). The results indicate that on a mass basis, the biosurfactants form micelles at lower concentrations than Tween 20, which is advantageous when applied to contaminated sites as the lower concentration of biosurfactant reduces the likelihood of toxic effects on soil microbes and also lowers the cost of remediation. Differences in CMCs of biosurfactants suggest that a lower concentration of saponin and higher concentration of sophorolipid are required to solubilize PAHs compared to rhamnolipid. Moreover, CMC of the extracted saponin biosurfactant on a mass basis is comparable with that of the crude saponin extracted from *Quillaja Saponaria* bark (30.2 mg/L) (Davin et al. 2018). In contrast, a purified saponin from the *Quillaja Saponaria* bark reported a much larger CMC in the range of 400–800 mg/L (Mitra and Dungan 1997). A larger CMC for the impure biosurfactants compared to the pure forms was previously reported (Jahan et al. 2020), while Hajimohammadi et al. (2016)reported a CMC of 150 mg/L for saponin extracted from *Glycyrrhiza glabra*.

### Comparison of the emulsifying ability and stability of biosurfactants in different environmental conditions

**Fig. 2:**
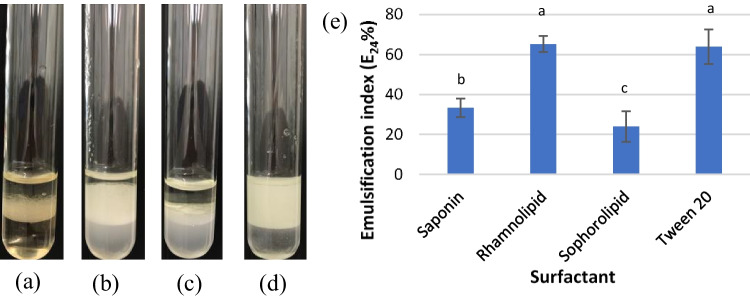
Emulsifying capacities (**a**: saponin, **b**: rhamnolipid, **c**: sophorolipid, and **d**: Tween 20) and emulsification indices of the surfactants (**e**). Different letters indicate significant differences among surfactants. Multiple comparisons of means were performed using one-way ANOVA with Tukey’s test at the 0.05 significance level

When aqueous surfactant solutions are mixed with a hydrophobic substance, they can induce an emulsion (Kabalnov 1998), which are kinetically stabilized, non-equilibrium systems. The ability of surfactants to produce stable emulsions under specific conditions can be determined by the emulsification index (Jahan et al. 2020).Emulsion-forming capacity and the stability of the emulsion formed by the rhamnolipid biosurfactant determined by the emulsification index were comparable to that of Tween 20,whilethe least stability was shown by sophorolipid at room temperature (Fig. 2).

**Fig. 3:**
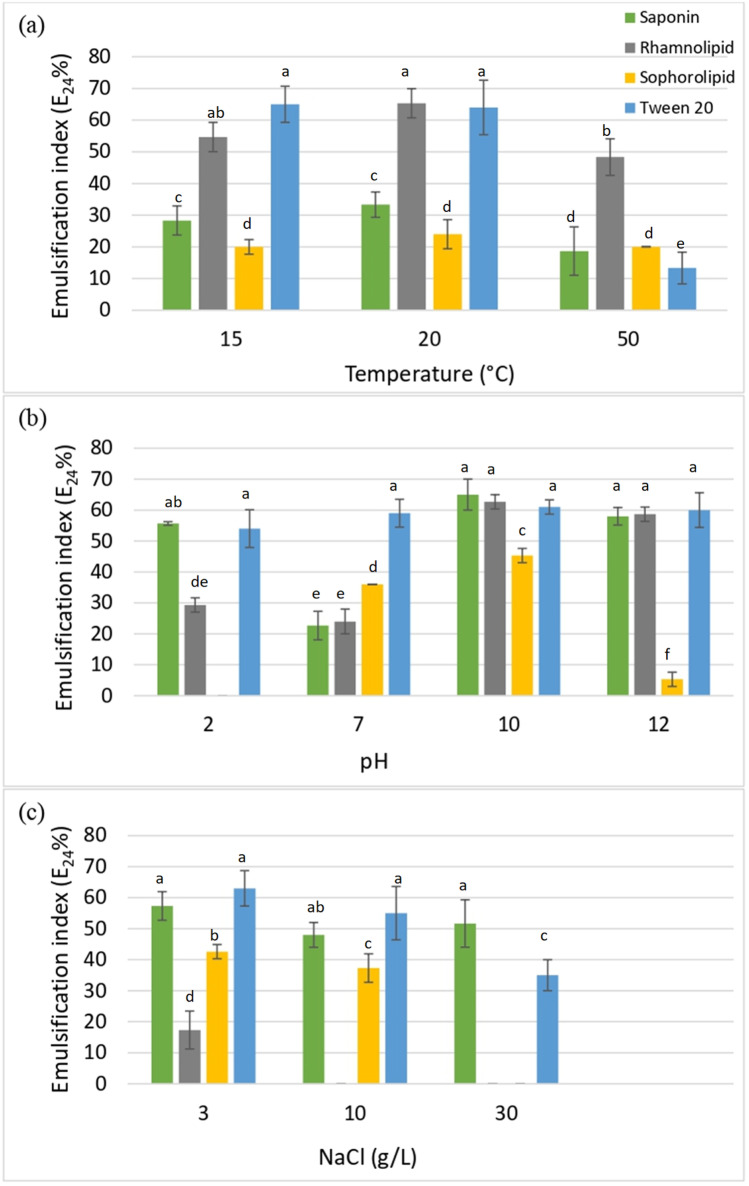
Stability of surfactants under different temperature (**a**), pH (**b**), and salinity (**c**) conditions. Different letters indicate significant differences among treatment conditions. Multiple comparisons of means were performed using one-way ANOVA with Tukey's test at the 0.05 significance level

The stability of biosurfactant emulsions and their physicochemical properties depend mainly on pH, temperature, and salinity (Jahan et al. 2020), and variation of these parameters may alter the self-assembly of micelles by changing their size or shape. The stability of the emulsions was determined based on the changes in the emulsification indices of the surfactants under different environmental conditions when compared to those at room temperature (20 °C). Changing the temperature from 15 to 20 °C did not significantly influence the stability of the emulsions formed by all the four surfactants (Fig. 3(a)). Increasing temperature resulted in slightly reducing the emulsion stability of saponin and rhamnolipid biosurfactants. The sophorolipid emulsion showed good thermal stability up to 50 °C. The Tween 20 emulsion revealed the highest reduction in stability at 50 °C. Accordingly, thermal stability of the emulsions formed by surfactants at the selected temperature range was in the order of sophorolipid > rhamnolipid > saponin > Tween 20. Despite the decrease in the emulsion stability, emulsions formed by all the biosurfactants were resistant to temperatures, ranging from 15–50 °C as they all maintained emulsifying activity across this temperature range and, therefore, were considered suitable for use on PAH-contaminated soils/sediments across a wide range of environmental temperatures.

Variation of the pH had no significant impact on the emulsifying action of Tween 20 (Fig. 3(b)) due to the non-ionic nature of the Tween 20 surfactant. The extracted saponin biosurfactant evinced a similar behavior to Tween 20, which may also be associated with the non-ionic nature of saponins (Rai et al. 2021).Among the biosurfactants studied, saponin denoted the highest stability under a wide range of acidic to basic pHs, and relatively higher stability was shown under acidic and basic compared to neutral pHs. The emulsification index of the anionic rhamnolipid in the basic pH medium was almost double the values in acidic and neutral media. When the carboxylate group is deprotonated under basic conditions, the rhamnolipid becomes more soluble in the aqueous phase, making the micelles more stable (Jahan et al. 2020). Jahan et al. (2020) and Kumar et al. (2012) also reported that alkaline pHs are optimum for rhamnolipid emulsions. The sophorolipid emulsion was only stable at pHs, ranging from 7 to 10. Sophorolipid may exist as anionic or non-ionic forms, which can alter the shape and self-assembly of micelles influencing the stability of the emulsion at low pHs (Baccile et al. 2012). As the average pH of soils and sediments lies between 6 and 7 (de Caritat et al. 2011; Herlihy and Mills 1986), sophorolipid may successfully be applied for PAH remediation. Compared to sophorolipid, relatively stable emulsions formed in the pH range, 2–12, indicating the applicability of rhamnolipid and saponin biosurfactants for remediation of PAH-contaminated soils/sediments over a wide range of pHs.

The salt concentration (3–30 g/L) had the least impact on the novel saponin biosurfactant emulsion (Fig. 3C). In contrast, rhamnolipid formed the least stable emulsion and was only stable at low saline conditions (3 g/L), demonstrating it is highly sensitive to the ionic strength (Jahan et al. 2020). The emulsion stability of sophorolipid was high in 3–10 g/L saline conditions but was shown to be unstable at 30 g/L. Tween 20 was as stable or more stable than the biosurfactants at 3–10 g/L salt concentration, but the emulsion stability reduced at higher concentrations. Accordingly, the order of tolerance up to 30 g/L salinity was saponin > Tween 20 > sophorolipid > rhamnolipid, which indicates that sophorolipid use would be restricted to soils with lower salinity, whereas the saponin biosurfactant could be applied to soils/sediments with more diverse salinity.

### Biosurfactant-aided desorption of  PAHs

Based on the PAH desorption efficiencies (Tables S2–S5) under the tested experimental conditions, optimized experimental conditions were determined and are presented in Table 2. In this study, surfactant concentrations of 0.5 × CMC, 1.25 × CMC, and 2 × CMC were used to obtain the best experimental conditions. Consequently, surfactant concentrations closest to their CMC were chosen, as that is the lowest concentration where micelles form and PAHs can be desorbed into the hydrophobic core. Use of low concentrations also minimizes their potentially toxic side effects to soil microbes and their potential to affects oil fertility(Shin et al. 2006).

**Table 2:** Optimized conditions for surfactant-mediated desorption of PAHs

Matrix	Surfactant	Experimental parameters		
		Concentration (mg/L)	Volume (mL)	Time (days)
Sediment	Saponin	2 × CMC	15	5
	Rhamnolipid	2 × CMC	15	5
	Sophorolipid	1.25 × CMC	8	5
	Tween 20	2 × CMC	10	5
Soil	Saponin	2 × CMC	10	5
	Rhamnolipid	1.25 × CMC	10	5
	Sophorolipid	1.25 × CMC	10	5
	Tween 20	2 × CMC	10	5

*CMC*, critical micelle concentration.

When the surfactant concentration was below the CMC (0.5 × CMC), only 10% desorption was observed, indicating that, under those conditions, very little of the PAHs were mobilized. On the other hand, when the concentrations were 1.25 × CMC and 2 × CMC, desorption was > 20%, indicating solubilization was more effective (Tables S2–S5). According to the results, optimum concentrations of the surfactants required to extract PAHs from soil and sediment were similar for saponin, sophorolipid, and Tween 20, whereas rhamnolipid required a lower concentration in soil than in sediment. Certain surfactants can form organic matter-biosurfactant complex micelles, which can enhance PAH removal in organic matter-rich matrices (Yu et al. 2014). If rhamnolipid has such ability, it may form complexes with organic matter when applied to the organic matter-rich soil (TOC—2.4%) compared to the sediment (TOC—0.2%).

Based on the results obtained from the response surface methodology, experimental conditions common for both soil and sediment were obtained for each surfactant. A surfactant concentration 2 × CMC was optimal for saponin, rhamnolipid, and Tween 20, whereas 1.25 × CMC was optimal for sophorolipid. Moreover, the surfactant volume of 15 mL was the best for saponin and rhamnolipid, and 10 mL was the ideal volume for sophorolipid and Tween 20.

**Fig. 4:**
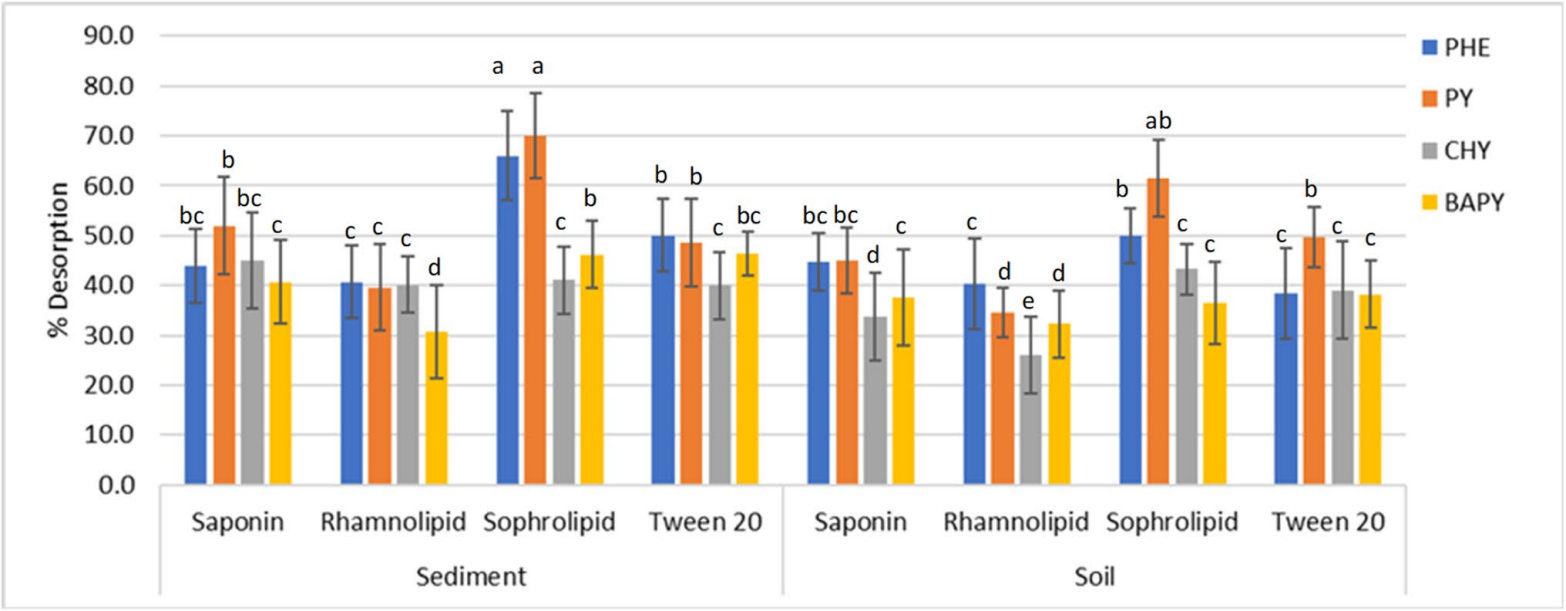
Surfactant-mediated desorption of PAHs (2 mg/kg) from soil and sediment. Different letters indicate significant differences among surfactant treatments. Multiple comparisons of means were performed using one- way ANOVA with Tukey's test at the 0.05 significance level

Figure 4 shows the PAH desorption efficiency of the surfactants when applied to spiked soil and sediment. All four surfactants demonstrated higher percentage desorptions of PHE and PY compared tothe more hydrophobic CHY and BAPY. Saponin and sophorolipid biosurfactants,respectively,showed 34–60% and 37–70% desorption of PAHs in sediment and soil,while Tween 20 and rhamnolipid were slightly less efficient with 26–41% and 38–50% desorptions, respectively. Bezza and Nkhalambayausi-Chirwa (2015) observed around 80% and 50% recovery of PHE and PY, respectively,in 8 days using the biosurfactant produced by *P. aeruginosa*, but they used surfactant at a rate of 4.7 × CMC, which undoubtedly improved the desorption efficiency. Furthermore, a rhamnolipid concentration of 100 × CMC resulted in 50–80% removal of PAHs in contaminated soil in 1 day at neutral pH and zero salinity (Zhang 2015). As noted by Zhang (2015), increasing biosurfactant concentration allows the surfactants to reach the maximum capacity of sorption to soil as the binding sites in soil become limited, which facilitates the formation of surfactant micelles. In contrast, Davin et al. (2018) determined a higher extraction efficiency of PAHs when the saponin concentration was 4 g/L compared to an 8-g/L solution, which was explained by the enhanced apparent sorption ofPAHs onto soil particles at high surfactant concentrations.However, the soil used was not sterilized, and potential microbial degradation of PAHs was not accounted for. Improved PAH extraction may not always occur at higher surfactant concentrations in all instances because the desorption capacity is dependent on other factors, including the nature of the matrix, other constituents presentin the surfactant solutions, and the experimental conditions. Thus, in the current study, low surfactant concentrations (60–120 mg/L) were chosen and shown to be effective in PAH desorption, as they are close to the respective biosurfactant CMCs.The results indicate that the PAH desorption efficiency of surfactants follows the order of sophorolipid > saponin = Tween 20 > rhamnolipid, and demonstrates their potential to improve the microbial degradation of PAHs, which does not appear to have been considered in other bioremediation studies to date.

Desorption of PAHs from sediment into the surfactant phase became less efficient at higher PAH concentrations, but this was not observed for soil (Tables S6 and S7). As sediment has less organic carbon, most PAHs might be mobilized by surfactants. Therefore, surfactant micelles may easily bind with sediment organic matter than PAHs (based on the affinity), as there are more binding sites available in organic matter. This may hinder the solubilization of the mobilized PAHs, or PAHs may re-associate with other constituents in the sediment/sediment-surfactant complexes. Desorption fell in sediment by 54% for PHE with saponin, 43% and 59% for CHY and BAPY, respectively, for rhamnolipid, and 46–47% for PY and BAPY using sophorolipid. Hence, increasing the volume of surfactant solution and incubation time can be recommended with increasing the dose of PAHs for efficient desorption.

Although there are studies conducted on biosurfactant-mediated removal of PAHs from soil, most of the researchers have used non-sterilized or bacteria-innoculated soils (Bezza and Chirwa 2016; Blyth et al. 2015; Bordas et al. 2005; Congiu and Ortega-Calvo 2014; Davin et al. 2018). Microbial degradation of PAHs has the potential to confound the results in studies where extraction occurs over several days, making soil sterilization an important part of studies such as these, because PAH half-lives may be as short as 10.5 days (Roslund et al. 2018).

PAHs can undergo oxidation and form oxy-PAHs during bioremediation due to chemical oxidation, photooxidation, or biological transformation (Staffan et al. 2007). Commonly found oxy-PAHs (Staffan et al. 2007) were not detected in the surfactant phase by GC–MS. This suggests that these compounds are not released to the environment during treatments. Oxy-PAHs are of major concern as these are carcinogenic and mutagenic to humans and more mobile than PAHs.

### Mass transport kinetics

**Fig. 5:**
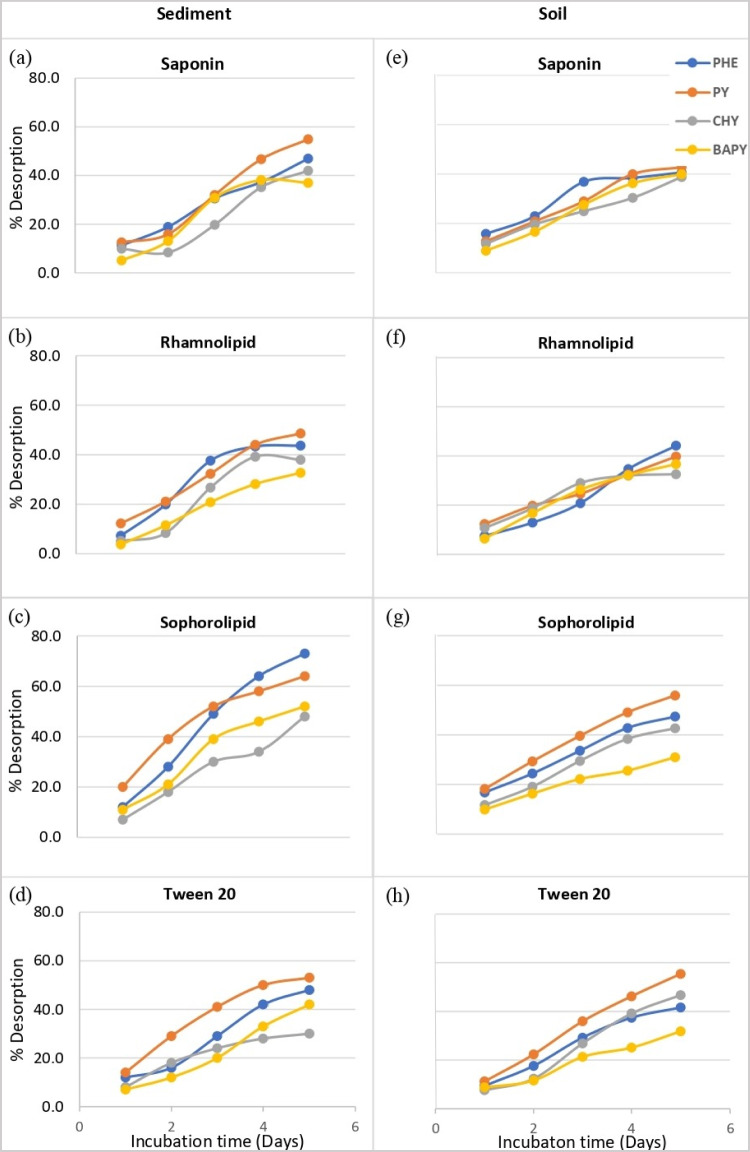
Surfactant-aided time-dependent desorption of PAHs (**a**, **b**, **c**, and **d** are for saponin, rhamnolipid, sophrolipid and Tween 20, respectively, in sediment; **e**, **f**, **g**, and **h** are for saponin, rhamnolipid, sophrolipid, and Tween 20, respectively, in soil)

The impact of time on the mass transfer of PAHs during the surfactant-aided desorption process was studied and shown in Fig. 5. Among the four surfactants, sophorolipid showed an initial rapid desorption of PAHs compared to the other surfactants. A relatively slower rate of mass transfer at the beginning of the experiment (1–2 days) was seen for PAHs in sediment than in soil. However, a slight reduction in the rate of desorption was observed after 3–4 days, mainly in sediment. It is important to note that the desorption of most of the PAHs did not plateau after the 5-day shaking period, and the PAH desorption continued after that, even at a lower rate of mass transfer. This shows that further desorption of PAHs can be expected by increasing the incubation time.

PAHs bound to readily accessible sorption sites can desorb rapidly and are considered the bioavailable fraction that may be microbially degraded. Surfactants can help desorb PAHs bound to less accessible sorption sites, increasing the concentration of bioavailable PAHs by incorporating them into the aqueous phase, where microbial degradation of PAHs mainly occurs (Bezza and Chirwa 2016), or by direct taken up by microbial absorption (Li and Chen 2009).Surfactants facilitate mass transfer by mobilization of PAHs at the concentrations below CMC and solubilization above the CMC (Bezza and Nkhalambayausi-Chirwa 2015).

In a previous report by Crampon et al. (2017), only 20% of PHE was desorbed by purified rhamnolipid solution after 8 days in one soil and 20 days for another soil sample, while over 90% removal was achieved after 20–40 days of incubation. Blyth et al. (2015)reported that a very long incubation period of 8 weeks was required to reduce the PAH concentration by 67%, and it was further reduced to 79% after 12 weeks, when biosurfactant extracted from the red ash tree was applied to historically contaminated soil. Bezza and Nkhalambayausi-Chirwa (2015), on the other hand, observed over 70% PHE desorption in 4 days in the presence of a crude lipopeptide produced by *P. aeruginosa*. Hence, longer incubation times, beyond the 5 days used in this study, may result in enhanced PAH desorption.

### Application of biosurfactants to aged soil and sediment and naturally incurred soil

Aging is the main factor that controls the PAH release mechanism in historically contaminated soils and sediments (Boulangé et al. 2019). However, few studies have investigated PAH-soil contact time (aging of soil) in biosurfactant-aided PAH desorption. Hence, desorption efficiency of PAHs in 30-day-aged spiked soil/sediment and naturally incurred soil was investigated. Studying spiked matrices aged for a short time period (30 days) can show differences in desorption efficiencies related to contact time between the PAHs and the matrices. Any differences may be related to changes in the reversibly bound PAHs, which occur at the surface of the soil/sediment particles due to sorption. Desorption efficiencies associated with the irreversible binding of PAHs, due to long-term sequestration in cores of soil particles, may be observed more in naturally incurred soils (Hamdi et al. 2012; Hatzinger and Alexander 1995). However, it is important to note that sorption and sequestration depend on the nature of the matrix constituents and the types of PAHs as binding energies and rates of associated sorption vary (Luthy et al. 1997).

**Fig. 6:**
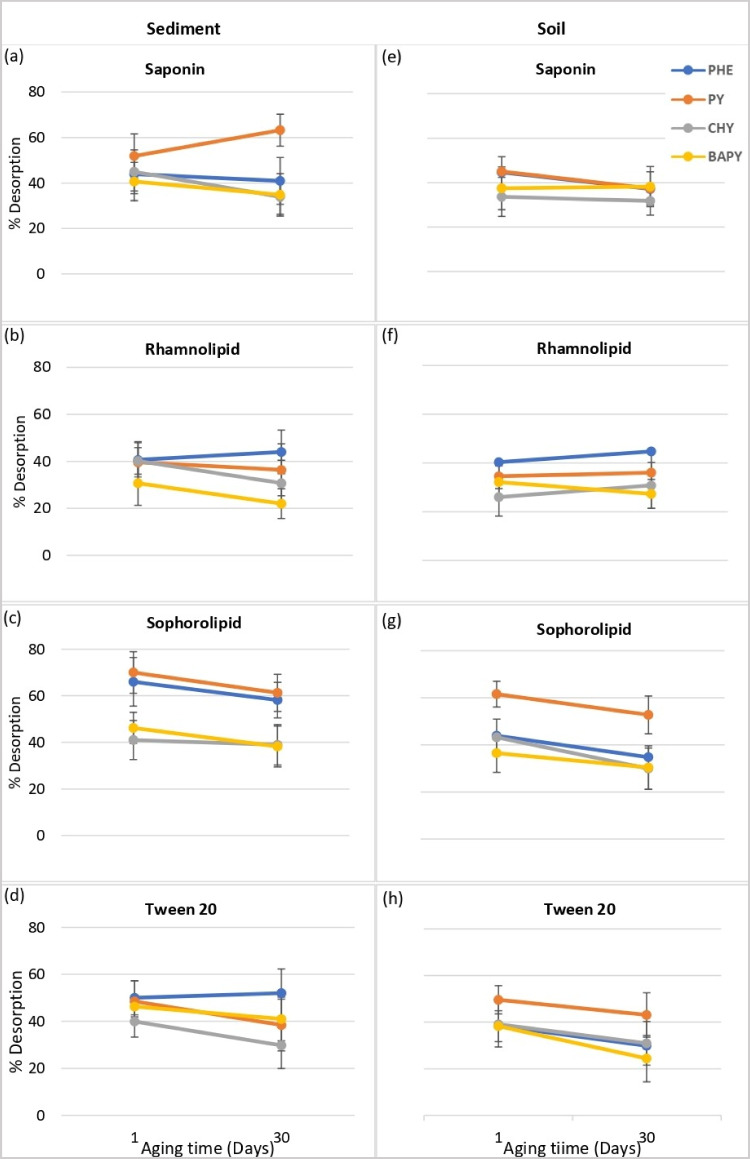
Effect of aging on surfactant-aided desorption of PAHs (**a**, **b**, **c**, and **d** are for saponin, rhamnolipid, sophorolipid, and Tween 20, respectively, in sediment; **e**, **f**, **g**, and **h** are for saponin, rhamnolipid, sophorolipid and Tween 20, respectively, in soil)

The results shown in Fig. 6 indicate there are no consistent trends across matrices, surfactants, or PAHs. However, the most common result was for desorption efficiency to decrease with aging. This is particularly noticeable for sophorolipid (Fig. 6(c) and Fig. 6(g)) and Tween 20 (Fig. 6(d) and Fig. 6(h)). Tween 20 showed the greatest reduction of PAH desorption both in aged soil and sediment compared to biosurfactants. Almost all PAHs in soil, as well as PY and CHY in sediment, showed a 20–40% decrease in desorption into the Tween 20 phase, and, therefore, Tween 20 was less efficient in PAH removal from aged soils/sediments than the biosurfactants. A decrease in desorption efficiency may be expected as the irreversibly bound desorption-resistant fraction may increase dramatically with the aging process (Hatzinger and Alexander 1995).

Desorption efficiencies for two of the biosurfactants—saponin (Fig. 6(a) and Fig. 6(e)) and rhamnolipid ( 6 (b) and Fig. 6(f) )—were least affected by the aging process, although there was some decrease in desorption efficiency for rhamnolipid extractions from sediment (Fig. 6(b)). It is not clear why the desorption efficiencies for these surfactants did not show the expected decrease. It may be that the changes in binding of the PAHs over time were such that these surfactants were still able to desorb the PAHs after 30 days of contact time. The complexity of the soil/sediment matrices, with different amounts of organic matter, makes further conclusions difficult; however, it may be noted that saponin and rhamnolipid are anionic surfactants, which may contribute to the results shown in Fig. 6.

**Fig. 7:**
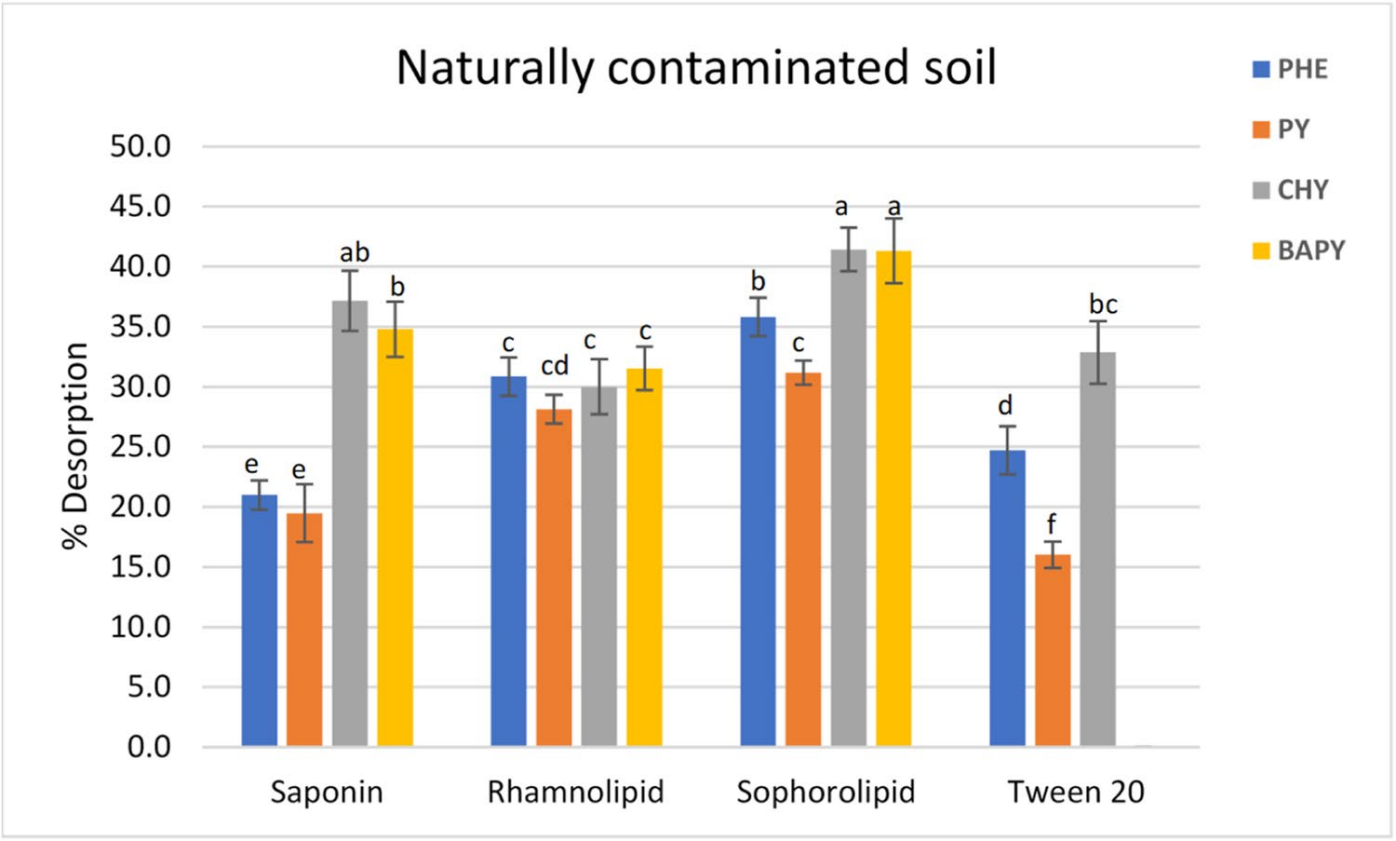
Surfactant-aided desorption of PAHs in naturally incurred soil. Different letters indicate significant differences among surfactant treatments. Multiple comparisons of means were performed using one-way ANOVA with Tukey’s test at the 0.05 significance level

The efficiencies of PAH removal by the biosurfactants were also studied for naturally incurred soil, as spiked soils do not mimic the naturally incurred soils (Wei et al. 2017).A naturally incurred soil used in a previous study (Kariyawasam et al. 2022) was found to have 0.81, 2.31, 0.7, and 0.92 mg/kg of PHE, PY, CHY, and BAPY, respectively, when quantified after eucalyptusoil-assisted extraction, and these were considered as the initial PAH concentrations in soil when calculating the percentage desorption in this study. As shown in Fig. 7, the percentage desorption of PHE, PY, CHY, and BAPY was 20–35%, 20–31%, 30–41%, and 31–41%, respectively, for the biosurfactants, whereas those for Tween 20 surfactant were 23% for PHE, 16%for PY, 33% for CHY, and 0% for BAPY.CHY showed the greatest desorption of the four PAHs tested. All the biosurfactants showed higher PAH removal than those of Tween 20, and the removal efficiencies followed the order of sophorolipid > rhamnolipid > saponin. Applications of saponin extracted from *Quillaja saponaria* bark (Davin et al. 2018) and rhamnolipid (Chong et al. 2014; Wang et al. 2014; Zhang et al. 2010) have been previously reported in field-contaminated soil. However, Davin et al. (2018) noted only 0.3–5% of PAH removal by saponin from soil in 24 h. According to Chong et al. (2014), 0.2–12% removal of LMW PAHs was noticed in 24 h with rhamnolipid, while Wang et al. (2014)reported 50–70% removal for 16 PAHs in 60 days. When the present study is compared with the above literature, considering the incubation times, studied biosurfactants incubated for 5 days seem to be comparably efficient in PAH removal. Furthermore, a gradual release of PAHs (“Mass transport kinetics” section) is important as this may prevent toxic effects to degrading microbes, or the release of PAHs into water bodies during the bioremediation process. This is the first study, to the best of the authors’ knowledge, that uses sophorolipid and eucalyptus saponin to remove PAHs in naturally contaminated soils. Among the studied surfactants, sophorolipid showed the most efficient PAH desorption behavior both in aged PAH-spiked soil/sediment and naturally incurred soil.

### The effect of surfactant amendment on soil microbial respiration

**Fig. 8:**
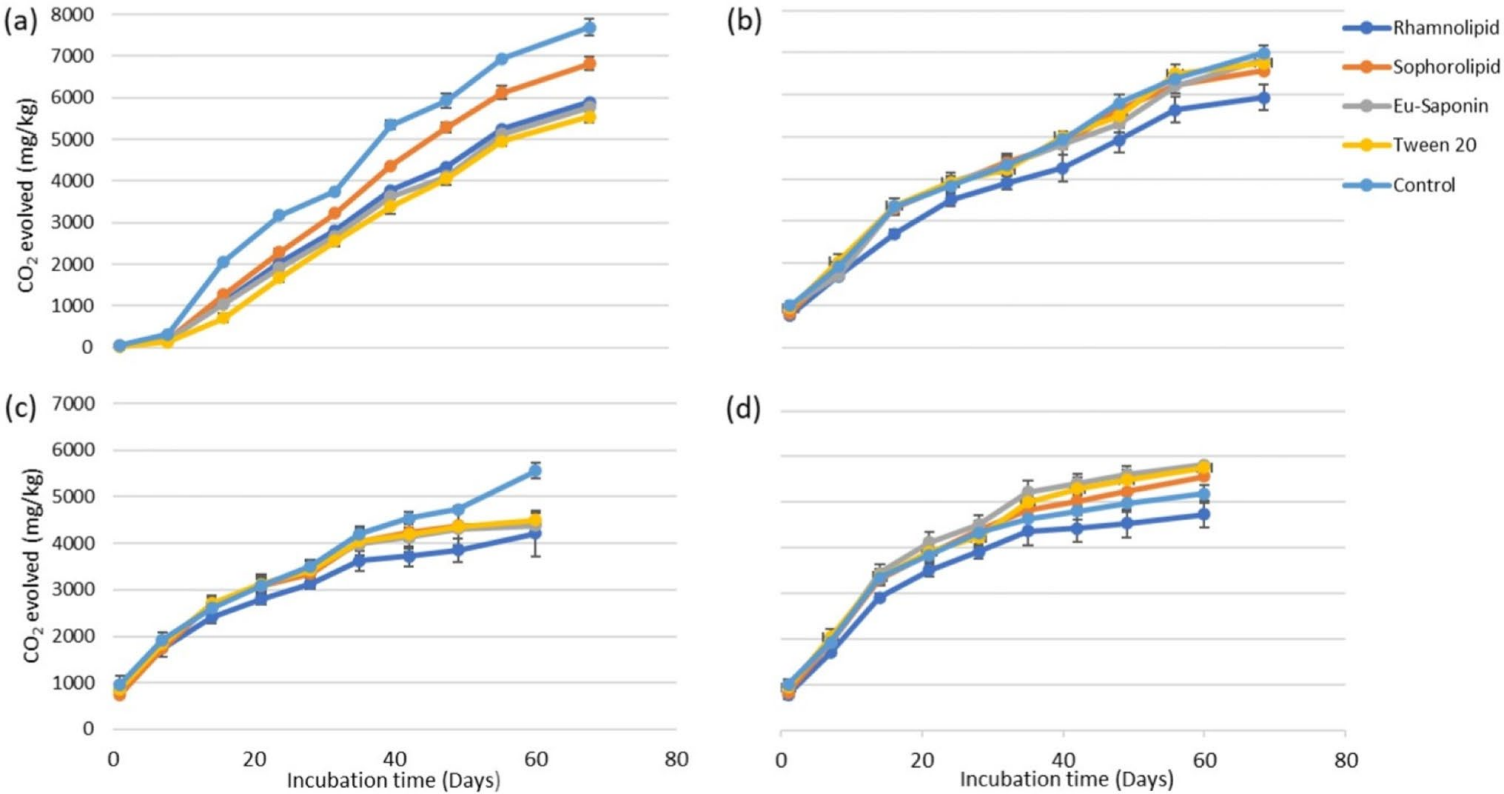
Effect of surfactant addition on CO_2_ evolution in **a**: PAH-free sediment, **b**: PAH-spiked sediment, **c**: PAH-free soil, and **d**: PAH-spiked soil

Moderate to non-toxic effects of biosurfactants have been detected for microbial species by determining microbial biomass, microbial respiration, enzyme activity, survival percentages, and by amplicon sequencing of bacterial genes to study the changes in bacterial structure and diversity (Souza et al. 2013; Edwards et al. 2003; Lu et al. 2019; Wolf and Gan 2018). In the presence of surfactants and PAHs, microbes may reduce their ability to mineralize carbon due to the inhibition of microbial activity (Nwachukwu and Pulford 2011). Therefore, the amount of CO_2_evolved is one of the effective indicators of the inhibitory effect of surfactant amendment on microbial activity. Potential toxic effects of surfactants on soil/sediment microbes were studied based on the amount ofCO_2_ evolved during microbial respiration (Fig. 8). Control samples demonstrate that the spiking of PAHs has not significantly (*P* < 0.05) influenced the microbial respiration in soil throughout the 60-day period. However, in sediment, a significantly (*P* < 0.05) higher amount of CO_2_ evolution was observed at the beginning in PAH-spiked samples compared to PAH-free samples.

In PAH-free sediment, all four surfactants and control showed a gradual increase in CO_2_ production until day 49, followed by slight retardation. Furthermore, in the presence of surfactants, a reduction in microbial respiration was detected in the samples compared to the control. However, total inhibition of microbial respiration was not observed over the 60-day period. Similar to the PAH-free sediment samples, the PAH-spiked samples showed a decline in CO_2_ evolution after day 49, while the degree of retardation was greater than in PAH-free sediment. After 60 days of incubation, microbial respiration in sophorolipid- and rhamnolipid- amended sediment was slightly higher than saponin and Tween 20 when PAHs were absent, whereas the opposite was true for the PAH-spiked samples. This observation signifies the impact of PAH-surfactant interaction on microbial respiration.

PAH-free soil in the presence of surfactants revealed a gradual increase in CO_2_ evolution, whereas the increment was not as steep as in sediment. Moreover, plateauing of the microbial respiration was observed after 35 days. Interactions of surfactant molecules with the higher amount of organic matter in the soil (12-fold) may have contributed to reduced microbial respiration compared to sediment. Notably, in the presence of PAHs, initial retardation in 14 days was identified with all the four surfactants, which was followed by a further decrease in the rate of microbial respiration after 35 days. As a comparable pattern was observed in PAH-free soil, a decrease in respiration could be due to the consumption of nutrients.

Results indicate that, in both soil and sediment, reduced microbial respiration was observed in the PAH-spiked rhamnolipid-amended samples. According to Lu et al. (2019), rhamnolipid has the ability to alter the microbial community composition and thus may influence microbial respiration. Nevertheless, microbial respiration was not completely inhibited in any of the samples during the 60-day incubation period. Hence, application of the surfactants to PAH-contaminated soil and sediment, even at a concentration of 500 mg/L, may not inhibit microbial activities. Further investigations are required to explore the individual and synergistic effects of different PAH and surfactant concentrations, temperature regimes, and various matrices to elucidate the toxicity of the selected surfactant on the soil/sediment microbiome. Although the effect of surfactant application on soil/sediment microbial respiration has been investigated (Memarian and Ramamurthy 2012), this is the first study done in the presence of PAHs and biosurfactants. The results shown in Fig. 8 are promising in that neither the biosurfactants alone nor in combination with PAHs were found to completely inhibit microbial respiration.

## Conclusion

Sophorolipid and extracted saponin biosurfactants exhibited efficient removal of PAHs in contaminated soils and sediments, comparable to the commonly applied rhamnolipid biosurfactant. The higher stability of emulsions derived from the saponin biosurfactant under a wide range of temperature, pH, and salinity conditions demonstrated its potential for PAH remediation under different environmental conditions. As observed, a gradual release of PAH into the aqueous phase was achieved, using low surfactant concentrations just above CMC, and that may reduce the potential toxic effect of rapid PAH desorption to soil/sediment microbes. Notably, the studied biosurfactants can be successfully applied to contaminated sites as inhibition of soil/sediment microbial respiration was not observed at the tested concentrations. Further research needs to focus on the effect of long-term incubation of biosurfactants with contaminated soils/sediments and toxic effects associated with higher surfactant concentrations and at different temperatures. PAH desorption capacity of biosurfactants in the presence of PAH-degrading microbes also needs further exploration.

## Supplementary Information

Below is the link to the electronic supplementary material.

Supplementary file1 (DOCX 172 KB)

## Data Availability

The datasets generated and/or analyzed during the current study are not publicly available but are available from the corresponding author on reasonable request.
