# Supplementary material for: Eucalyptus saponin- and sophorolipid-mediated desorption of polycyclic aromatic hydrocarbons from contaminated soil and sediment
Source: Environ Sci Pollut Res Int. 2022 Oct 22;30(8):21638–53. doi: 10.1007/s11356-022-23562-z (PMC9938058; doi:10.1007/s11356-022-23562-z)
Supplement: Supplementary file 1 — Supplementary file1 (DOCX 172 KB) [file 11356_2022_23562_MOESM1_ESM.docx]

**Supporting Information**

**Eucalyptus saponin- and sophorolipid-mediated desorption of polycyclic aromatic hydrocarbons from contaminated soil and sediment**

Thiloka Kariyawasam^ab^, Paul D. Prenzler^ab^, Julia A. Howitt^ab^, Gregory S. Doran^ab^*

^a^School of Agricultural, Environmental and Veterinary Sciences, Charles Sturt University, Wagga Wagga, NSW, 2678, Australia

^b^Gulbali Institute, Charles Sturt University, Wagga Wagga, NSW, 2678, Australia

***Corresponding Author-** Email [gdoran@csu.edu.au](mailto:gdoran@csu.edu.au)


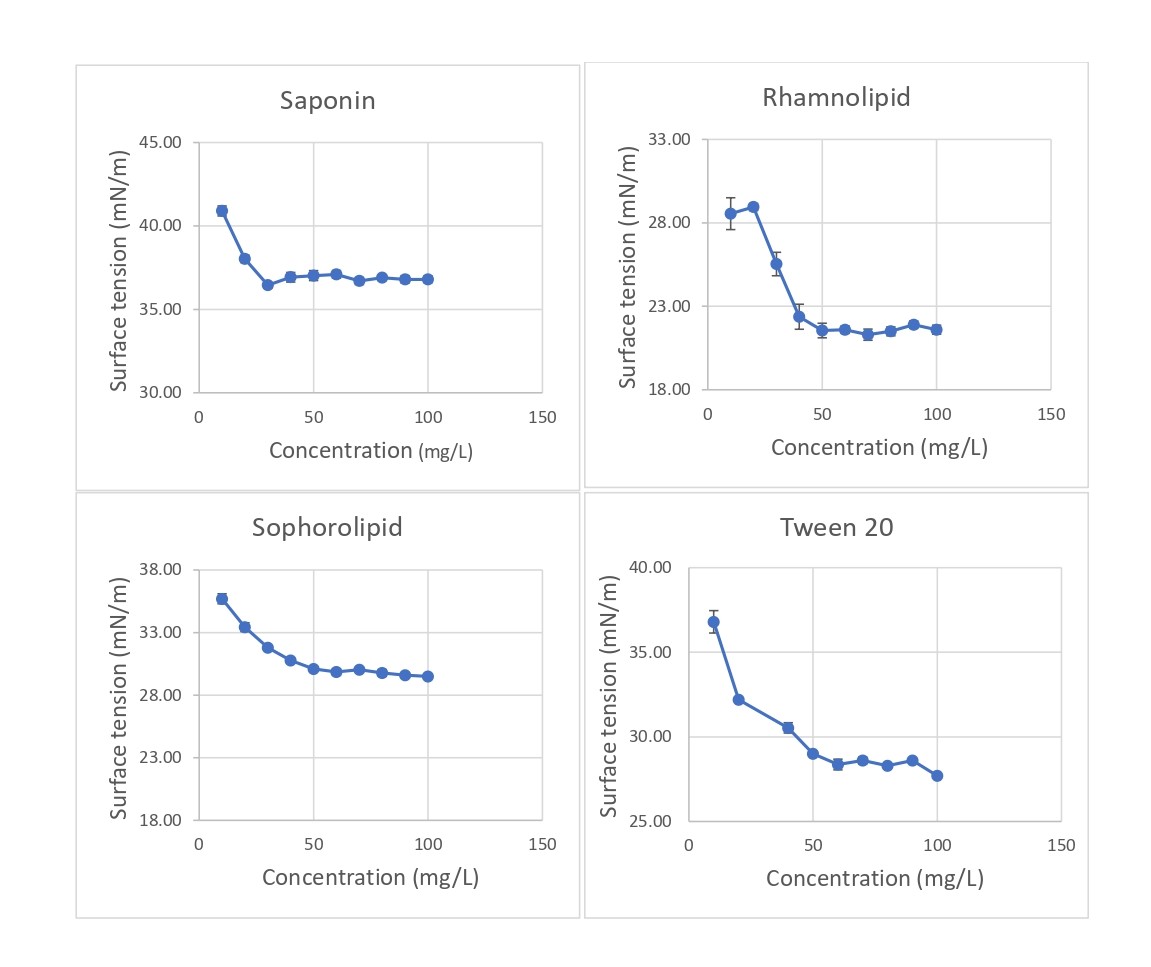


Fig. S1 Surface tension of the aqueous surfactant solutions at 20 °C, measured according to the Wilhelmy Plate method

Table S1 Box–Behnken experimental design for surfactant aided desorption of PAHs

| Concentration (mg/L) | Volume (mL) | Incubation period (Days) |
| --- | --- | --- |
|  |  |  |
| 1.25×CMC | 10 | 5 |
| 1.25×CMC | 5 | 7 |
| 1.25×CMC | 10 | 5 |
| 0.5×CMC | 10 | 7 |
| 0.5×CMC | 15 | 5 |
| 2×CMC | 5 | 5 |
| 0.5×CMC | 15 | 5 |
| 2×CMC | 10 | 3 |
| 1.25×CMC | 10 | 5 |
| 0.5×CMC | 10 | 3 |
| 0.5×CMC | 5 | 5 |
| 1.25×CMC | 15 | 7 |
| 2×CMC | 10 | 3 |
| 1.25×CMC | 10 | 5 |
| 0.5×CMC | 10 | 3 |
| 1.25×CMC | 5 | 3 |
| 2×CMC | 10 | 7 |
| 1.25×CMC | 5 | 7 |
| 2×CMC | 5 | 5 |
| 1.25×CMC | 10 | 5 |
| 1.25×CMC | 15 | 3 |
| 0.5×CMC | 10 | 7 |
| 2×CMC | 15 | 5 |
| 1.25×CMC | 10 | 5 |
| 0.5×CMC | 5 | 5 |
| 1.25×CMC | 15 | 3 |
| 2×CMC | 15 | 5 |
| 1.25×CMC | 5 | 3 |
| 1.25×CMC | 15 | 7 |
| 2×CMC | 10 | 7 |

| Concentration (mg/L) | Volume (mL) | Incubation period (Days) | Percentage desorption (%) | | | |
| --- | --- | --- | --- | --- | --- | --- |
|  |  |  | PHE | PY | CHY | BAPY |
| Sediment | | | | | | |
| 1.25×CMC | 10 | 5 | 40.3 ± 4.8 | 55.7 ± 4.3 | 41.9 ± 5.3 | 40.3 ± 3.4 |
| 1.25×CMC | 5 | 7 | 24.6 ± 5.2 | 36.8 ± 5.5 | 33.4 ± 5.2 | 24.2 ± 4.2 |
| 0.5×CMC | 15 | 5 | 16.2 ± 3.6 | 19.4 ± 3.4 | 16.3 ± 3.5 | 12.1 ± 1.6 |
| 2×CMC | 5 | 5 | 25.4 ± 5.1 | 38.9 ± 2.9 | 30.7 ± 5.3 | 25.1 ± 4 |
| 2×CMC | 10 | 3 | 28.7 ± 1.7 | 41.3 ± 4.2 | 33.6 ± 5.1 | 27.3 ± 1.4 |
| 1.25×CMC | 15 | 7 | 33.1 ± 4 | 43.4 ± 1.8 | 38.6 ± 2.3 | 33.0 ± 4 |
| 0.5×CMC | 10 | 3 | 13.1 ± 1.9 | 14.6 ± 3.3 | 10.2 ± 1.9 | 9.1 ± 1.6 |
| 1.25×CMC | 5 | 3 | 24.2 ± 2.3 | 31.3 ± 3.4 | 27.3 ± 5.1 | 20.8 ± 7.1 |
| 2×CMC | 10 | 7 | 38.3 ± 1.4 | 53.2 ± 6.2 | 41.7 ± 4.5 | 37.4 ± 4.5 |
| 1.25×CMC | 15 | 3 | 30.7 ± 3.8 | 40.6 ± 2.1 | 35.2 ± 6.1 | 31.4 ± 7.6 |
| 0.5×CMC | 10 | 7 | 14.7 ± 2.4 | 16.1 ± 1.8 | 12.3 ± 1.7 | 8.2 ± 0.6 |
| 0.5×CMC | 5 | 5 | 9.5 ± 0.6 | 10.7 ± 1.0 | 7.3 ± 0.5 | 6.8 ± 0.9 |
| 2×CMC | 15 | 5 | 46.2 ± 4.1 | 63.8 ± 2.4 | 46.7 ± 3.2 | 48.0 ± 2.4 |
| Soil | | | | | | |
| 1.25×CMC | 10 | 5 | 36.1 ± 5.2 | 36.3 ± 5.5 | 27.2 ± 1.2 | 30.1 ± 1.8 |
| 1.25×CMC | 5 | 7 | 22.1 ± 2.3 | 18.6 ± 1.4 | 16.8 ± 0.9 | 16.1 ± 1.3 |
| 0.5×CMC | 15 | 5 | 12.3 ± 1.8 | 10.1 ± 1.1 | 7.1 ± 0.4 | 7.4 ± 0.5 |
| 2×CMC | 5 | 5 | 40.7 ± 4.6 | 41.3 ± 3.4 | 30.3 ± 3.2 | 35.0 ± 3.1 |
| 2×CMC | 10 | 3 | 45.8 ± 1.2 | 46.7 ± 3.7 | 36.4 ± 2.7 | 39.8 ± 3.4 |
| 1.25×CMC | 15 | 7 | 27.1 ± 0.9 | 25.0 ± 4.7 | 20.7 ± 3.9 | 18.4 ± 3.5 |
| 0.5×CMC | 10 | 3 | 14.1 ± 1.5 | 12.8 ± 1.7 | 8.4 ± 0.8 | 9.5 ± 0.3 |
| 1.25×CMC | 5 | 3 | 23.1 ± 3.8 | 20.3 ± 3.5 | 16.2 ± 1.9 | 18.4 ± 1.2 |
| 2×CMC | 10 | 7 | 43.9 ± 7.1 | 44.7 ± 1.9 | 34.1 ± 2.6 | 36.4 ± 4.5 |
| 1.25×CMC | 15 | 3 | 31.3 ± 6.1 | 30.2 ± 3.6 | 25.1 ± 4.2 | 22.3 ± 4.6 |
| 0.5×CMC | 10 | 7 | 9.3 ± 0.9 | 9.4 ± 0.7 | 6.0 ± 0.4 | 5.1 ± 0.5 |
| 0.5×CMC | 5 | 5 | 9.1 ± 1.2 | 9.8 ± 1.1 | 6.3 ± 0.4 | 5.7 ± 0.4 |
| 2×CMC | 15 | 5 | 43.2 ± 5.8 | 45.3 ± 1.3 | 34.4 ± 3.6 | 38.2 ± 3.6 |

Table S2 Percentage desorption of PAHs by saponin under different experimental conditions

Table S3 Percentage desorption of PAHs by rhamnolipid under different experimental conditions

| Concentration (mg/L) | Volume (mL) | Incubation period (Days) | Percentage desorption (%) | | | |
| --- | --- | --- | --- | --- | --- | --- |
|  |  |  | PHE | PY | CHY | BAPY |
| Sediment | | | | | | |
| 1.25×CMC | 10 | 5 | 38.7± 3.7 | 40.4 ± 5.1 | 38.4 ± 2.9 | 33.3 ± 4.3 |
| 1.25×CMC | 5 | 7 | 28.4 ± 5.7 | 33.5 ± 4.3 | 28.3 ± 3.9 | 22.0 ± 3.7 |
| 0.5×CMC | 15 | 5 | 19.2 ± 1.4 | 17.2 ± 1.5 | 14.3 ± 1.1 | 11.0 ± 0.9 |
| 2×CMC | 5 | 5 | 30.3 ± 2.1 | 33.2 ± 4.1 | 32.4 ± 4.2 | 24.1 ± 2.2 |
| 2×CMC | 10 | 3 | 34.3 ± 5.9 | 36.5 ± 3.1 | 36.2 ± 2.6 | 30.3 ± 3.6 |
| 1.25×CMC | 15 | 7 | 36.4 ± 1.1 | 38.9 ± 2.7 | 37.1 ± 2.5 | 32.1 ± 2 |
| 0.5×CMC | 10 | 3 | 11.7 ± 0.6 | 13.6 ± 1.5 | 8.0 ± 1.1 | 7.3 ± 0.3 |
| 1.25×CMC | 5 | 3 | 25.2 ± 5.6 | 30.3 ± 3.4 | 22.7 ± 4.2 | 17.5 ± 5.2 |
| 2×CMC | 10 | 7 | 43.4 ± 4.3 | 48.2 ± 4.1 | 37.5 ± 1.3 | 36.7 ± 4.6 |
| 1.25×CMC | 15 | 3 | 33.9 ± 4.6 | 35.8 ± 3.2 | 36.0 ± 4.6 | 28.5 ± 9 |
| 0.5×CMC | 10 | 7 | 15.4 ± 1.8 | 11.2 ± 1.1 | 11.6 ± 3.2 | 9.8 ± 0.7 |
| 0.5×CMC | 5 | 5 | 10.1 ± 1.8 | 10.0 ± 0.4 | 7.2 ± 0.6 | 4.3 ± 1.8 |
| 2×CMC | 15 | 5 | 41.3 ± 2.7 | 54.4 ± 1.5 | 45.3 ± 5.3 | 39.7 ± 7.6 |
| Soil | | | | | | |
| 1.25×CMC | 10 | 5 | 47.2 ± 3.8 | 43.9 ± 5 | 31.4 ± 3.7 | 37.9 ± 5.2 |
| 1.25×CMC | 5 | 7 | 35.1 ± 4.4 | 31.7 ± 3 | 20.3 ± 2.5 | 28.8 ± 4.2 |
| 0.5×CMC | 15 | 5 | 20.7 ± 2.7 | 15.4 ± 1.4 | 10.2 ± 0.6 | 15.1 ± 1.6 |
| 2×CMC | 5 | 5 | 40.1 ± 3.8 | 35.1 ± 3.3 | 27.4 ± 3.5 | 31.5 ± 4.3 |
| 2×CMC | 10 | 3 | 41.7 ± 5.1 | 34.7 ± 5.9 | 25.9 ± 4.6 | 30.3 ± 2 |
| 1.25×CMC | 15 | 7 | 35.8 ± 4.2 | 31.9 ± 3.2 | 23.4 ± 4.3 | 35.4 ± 4.7 |
| 0.5×CMC | 10 | 3 | 17.2 ± 1.3 | 11.1 ± 1.2 | 8.4 ± 0.8 | 13.2 ± 0.6 |
| 1.25×CMC | 5 | 3 | 41.4 ± 3.7 | 36.6 ± 3.1 | 26.1 ± 3.5 | 33.7 ± 4.1 |
| 2×CMC | 10 | 7 | 33.5 ± 2.5 | 35.3 ± 4.4 | 28.7 ± 4.1 | 33.6 ± 2.7 |
| 1.25×CMC | 15 | 3 | 45.1 ± 1.6 | 38.7 ± 4.8 | 25.1 ± 4.2 | 36.3 ± 1.9 |
| 0.5×CMC | 10 | 7 | 10.7 ± 0.5 | 7.2 ± 1.1 | 10.2 ± 1.3 | 11.6 ± 1.4 |
| 0.5×CMC | 5 | 5 | 9.8 ± 0.6 | 7.7 ± 0.2 | 8.6 ± 1.2 | 8.1 ± 1.1 |
| 2×CMC | 15 | 5 | 35.3 ± 3 | 31.2 ± 4.2 | 22.4 ± 4.2 | 27.6 ± 4.1 |

Table S4 Percentage desorption of PAHs by sophorolipid under different experimental conditions

| Concentration (mg/L) | Volume (mL) | Incubation period (Days) | Percentage desorption (%) | | | |
| --- | --- | --- | --- | --- | --- | --- |
|  |  |  | PHE | PY | CHY | BAPY |
| Sediment | | | | | | |
| 1.25×CMC | 10 | 5 | 62.8 ± 4.6 | 68.9 ± 3.8 | 40.5 ± 3.4 | 56.4 ± 4.7 |
| 1.25×CMC | 5 | 7 | 49.6 ± 7 | 45.5 ± 4.6 | 22.7 ± 2.1 | 32.9 ± 5.7 |
| 0.5×CMC | 15 | 5 | 23.2 ± 1.7 | 20.6 ± 1.2 | 14.1 ± 0.9 | 12.6 ± 0.5 |
| 2×CMC | 5 | 5 | 54 ± 4.2 | 50.2 ± 0.9 | 27.4 ± 1.1 | 38.1 ± 1.9 |
| 2×CMC | 10 | 3 | 54.1 ± 1.9 | 54.7 ± 3.5 | 30.3 ± 2.6 | 42.7 ± 1.9 |
| 1.25×CMC | 15 | 7 | 45.7 ± 2.3 | 40.1 ± 3.8 | 21.3 ± 5.6 | 28.5 ± 2.4 |
| 0.5×CMC | 10 | 3 | 18.3 ± 1.2 | 15.4 ± 1.1 | 12 ± 1.3 | 9.7 ± 0.5 |
| 1.25×CMC | 5 | 3 | 56.2 ± 3.8 | 60.1 ± 6.1 | 33.4 ± 4.6 | 46.1 ± 3.6 |
| 2×CMC | 10 | 7 | 57.3 ± 4.4 | 58.9 ± 4.9 | 34.7 ± 4.8 | 44.5 ± 5.7 |
| 1.25×CMC | 15 | 3 | 58.3 ± 2.6 | 64.2 ± 3.8 | 37.3 ± 5.8 | 50.7 ± 2.1 |
| 0.5×CMC | 10 | 7 | 12.2 ± 1 | 11.7 ± 1.8 | 9.2 ± 0.7 | 8.1 ± 1.3 |
| 0.5×CMC | 5 | 5 | 11.5 ± 1.2 | 9.6 ± 0.7 | 7.2 ± 1.8 | 7.4 ± 1.1 |
| 2×CMC | 15 | 5 | 44.3 ± 4.3 | 42.6 ± 5.7 | 22.7 ± 4.4 | 25 ± 4.3 |
| Soil | | | | | | |
| 1.25×CMC | 10 | 5 | 49.1 ± 4.5 | 56.8 ± 3.2 | 42.7 ± 3.6 | 33.6 ± 2.4 |
| 1.25×CMC | 5 | 7 | 28.6 ± 4.1 | 37.8 ± 1.2 | 25.3 ± 2.9 | 19.8 ± 1.5 |
| 0.5×CMC | 15 | 5 | 17.4 ± 1.1 | 16.9 ± 0.4 | 12.7 ± 0.9 | 11.4 ± 1.5 |
| 2×CMC | 5 | 5 | 38.7 ± 2.7 | 47 ± 5.4 | 34.8 ± 4.1 | 25.3 ± 3 |
| 2×CMC | 10 | 3 | 39.3 ± 3.4 | 46.7 ± 3.2 | 34.4 ± 4.2 | 27.2 ± 5.4 |
| 1.25×CMC | 15 | 7 | 33.2 ± 4.1 | 42.4 ± 2.7 | 30.1 ± 2.6 | 22.4 ± 3.3 |
| 0.5×CMC | 10 | 3 | 12.1 ± 1.4 | 11.7 ± 0.9 | 9.4 ± 1.5 | 9.1 ± 0.9 |
| 1.25×CMC | 5 | 3 | 40.5 ± 3.5 | 48.2 ± 3.8 | 35.9 ± 3.1 | 26.5 ± 3.2 |
| 2×CMC | 10 | 7 | 44.8 ± 3.4 | 49.8 ± 2.9 | 36.9 ± 6.2 | 27.2 ± 2 |
| 1.25×CMC | 15 | 3 | 44.3 ± 4.1 | 51.7 ± 2.6 | 38.3 ± 1.3 | 30.2 ± 1 |
| 0.5×CMC | 10 | 7 | 9.7 ± 0.7 | 9.1 ± 1.2 | 7.4 ± 0.9 | 6.2 ± 0.7 |
| 0.5×CMC | 5 | 5 | 8.6 ± 3.1 | 8 ± 3.4 | 5.3 ± 3.2 | 5.4 ± 4.8 |
| 2×CMC | 15 | 5 | 33.9 ± 4.3 | 43.7 ± 3.2 | 31.8 ± 2.2 | 21.7 ± 4.1 |

Table S5 Percentage desorption of PAHs by Tween 20 under different experimental conditions

| Concentration (mg/L) | Volume (mL) | | Incubation period (Days) | Percentage desorption (%) | | | | | | |
| --- | --- | --- | --- | --- | --- | --- | --- | --- | --- | --- |
|  |  |  |  | PHE | | PY | | CHY | | BAPY |
| Sediment | | | | | | | | | | |
| 1.25×CMC | 10 | 5 | | 40.4 ± 5.3 | 41.2 ± 9.6 | | 30.7 ± 1.8 | | 32.4 ± 3.4 | |
| 1.25×CMC | 5 | 7 | | 26.4 ± 3.7 | 24.7 ± 9 | | 14.5 ± 3.6 | | 15.4 ± 3.1 | |
| 0.5×CMC | 15 | 5 | | 12.4 ± 2.5 | 13.7 ± 1.7 | | 7.7 ± 1.2 | | 9.5 ± 1.1 | |
| 2×CMC | 5 | 5 | | 43.7 ± 1.6 | 45.9 ± 4 | | 33.1 ± 8.3 | | 38.5 ± 4.5 | |
| 2×CMC | 10 | 3 | | 45.6 ± 3.5 | 50.1 ± 1 | | 36.5 ± 3.6 | | 40.7 ± 3 | |
| 1.25×CMC | 15 | 7 | | 35.5 ± 4.6 | 33.1 ± 2.2 | | 22.5 ± 2 | | 21.6 ± 5.2 | |
| 0.5×CMC | 10 | 3 | | 17.3 ± 3 | 17.7 ± 1.4 | | 10.6 ± 4.1 | | 10.1 ± 4.2 | |
| 1.25×CMC | 5 | 3 | | 31.3 ± 4.8 | 29.8 ± 4 | | 18.7 ± 2.5 | | 17.9 ± 4.2 | |
| 2×CMC | 10 | 7 | | 44.7 ± 3.5 | 48.3 ± 3.6 | | 35.2 ± 7.6 | | 40.4 ± 9.4 | |
| 1.25×CMC | 15 | 3 | | 38.4 ± 4.1 | 36.9 ± 3.5 | | 27.7 ± 9.2 | | 26.3 ± 4.2 | |
| 0.5×CMC | 10 | 7 | | 8.4 ± 2.2 | 7.4 ± 9.1 | | 4.8 ± 1.8 | | 6.3 ± 1.9 | |
| 0.5×CMC | 5 | 5 | | 9.3 ± 1.3 | 8 ± 1.7 | | 6.5 ± 1.3 | | 7.1 ± 1.2 | |
| 2×CMC | 15 | 5 | | 44 ± 2.9 | 47.4 ± 2.4 | | 34.8 ± 3.5 | | 41.1 ± 3.6 | |
| Soil | | | | | | | | | | |
| 1.25×CMC | 10 | 5 | | 34.2 ± 2.7 | 40.7 ± 1.8 | | 31.3 ± 5.1 | | 20 ± 5.6 | |
| 1.25×CMC | 5 | 7 | | 19.5 ± 3.8 | 22.6 ± 4.3 | | 16.9 ± 4.5 | | 10.2 ± 2.3 | |
| 0.5×CMC | 15 | 5 | | 12.1 ± 2.1 | 13.9 ± 3.4 | | 9.2 ± 2.1 | | 8.7 ± 2 | |
| 2×CMC | 5 | 5 | | 37.1 ± 4.2 | 44.7 ± 5.5 | | 35.9 ± 5.3 | | 22.7 ± 4.7 | |
| 2×CMC | 10 | 3 | | 45.8 ± 5.3 | 54 ± 3.4 | | 44.1 ± 5.2 | | 30.4 ± 4.6 | |
| 1.25×CMC | 15 | 7 | | 26.6 ± 3.7 | 31.5 ± 2.9 | | 22.3 ± 3.5 | | 13.8 ± 4.1 | |
| 0.5×CMC | 10 | 3 | | 14.1 ± 2.5 | 15.7 ± 4.2 | | 11.3 ± 1.3 | | 8.4 ± 1.7 | |
| 1.25×CMC | 5 | 3 | | 22.4 ± 1.6 | 26.2 ± 3.2 | | 18.7 ± 4.2 | | 11.1 ± 1.3 | |
| 2×CMC | 10 | 7 | | 43.2 ± 3.5 | 50.3 ± 6.2 | | 41.5 ± 2.3 | | 27.2 ± 5.6 | |
| 1.25×CMC | 15 | 3 | | 30.1 ± 4.6 | 36.9 ± 2.1 | | 27.2 ± 3.9 | | 16.3 ± 2.2 | |
| 0.5×CMC | 10 | 7 | | 8 ± 0.8 | 7.5 ± 1.2 | | 6.1 ± 0.9 | | 7.1 ± 1.7 | |
| 0.5×CMC | 5 | 5 | | 9.8 ± 1.1 | 9.1 ± 1.8 | | 7.9 ± 1.0 | | 8.3 ± 1.2 | |
| 2×CMC | 15 | 5 | | 40.3 ± 5.2 | 48.6 ± 3.7 | | 40.6 ± 2.7 | | 25.8 ± 3.1 | |

Table S6 Desorption efficiencies of PAHs in sediment

| Surfactant | Concentration of PAH (mg/kg of sediment) | Percentage desorption (%) | | | |
| --- | --- | --- | --- | --- | --- |
|  |  | PHE | PY | CHY | BAPY |
| Saponin | 0.5 | 65 ± 9.6 | 53.7 ± 1.8 | 36.5 ± 3.8 | 41.7 ± 3.4 |
|  | 1 | 51.3 ± 9 | 67.3 ± 7.6 | 48.9 ± 3.4 | 42.4 ± 3.1 |
|  | 2 | 47.4 ± 1.7 | 68.9 ± 5.2 | 47.2 ± 4.7 | 56 ± 7.1 |
|  | 5 | 30.1 ± 4 | 73 ± 8.3 | 42.3 ± 4.6 | 53.4 ± 4.5 |
|  | 10 | 21.9 ± 1 | 64.8 ± 3.6 | 39.5 ± 2.1 | 36.4 ± 3 |
| Rhamnolipid | 0.5 | 29.2 ± 2.2 | 20.4 ± 2 | 19.3 ± 5.7 | 38.1 ± 5.2 |
|  | 1 | 41.9 ± 1.4 | 52.1 ± 7.1 | 29.9 ± 1.2 | 15.4 ± 4.6 |
|  | 2 | 43.2 ± 4 | 62.2 ± 4.5 | 48 ± 5.9 | 35 ± 6.2 |
|  | 5 | 40.2 ± 4.6 | 49.5 ± 8.2 | 33.4 ± 3.5 | 19.8 ± 2 |
|  | 10 | 32.5 ± 2.6 | 48.9 ± 1.3 | 27.2 ± 0.9 | 14.5 ± 1 |
| Sophrolipid | 0.5 | 68.2 ± 7.1 | 40.5 ± 6 | 27.6 ± 1.1 | 25.7 ± 7.4 |
|  | 1 | 60.5 ± 4.8 | 35.3 ± 2 | 27.6 ± 1.9 | 43 ± 4.8 |
|  | 2 | 72.7 ± 3.6 | 60.7 ± 7.6 | 40.3 ± 3 | 64.6 ± 9.4 |
|  | 5 | 70.1 ± 3.5 | 44.2 ± 9.2 | 34.5 ± 1.2 | 43.1 ± 4.2 |
|  | 10 | 70.1 ± 9.1 | 32.6 ± 1.8 | 25.5 ± 9 | 34.3 ± 1.9 |
| Tween 20 | 0.5 | 21 ± 2.7 | 20.4 ± 3 | 10.1 ± 5.4 | 15.3 ± 4.2 |
|  | 1 | 39.3 ± 1.8 | 51.4 ± 5.1 | 27.4 ± 3.2 | 25.4 ± 5.6 |
|  | 2 | 44.2 ± 4.3 | 55.4 ± 4.5 | 39.5 ± 5.7 | 43 ± 4.3 |
|  | 5 | 48.3 ± 3.4 | 42.1 ± 6.1 | 33.8 ± 3.9 | 47.2 ± 2 |
|  | 10 | 33.6 ± 5.5 | 40.2 ± 5.3 | 33.2 ± 3.8 | 44.5 ± 4.7 |

Table S7 Desorption efficiencies of PAHs in soil

| Surfactant | Concentration of PAH (mg/kg of sediment) | Percentage desorption (%) | | | |
| --- | --- | --- | --- | --- | --- |
|  |  | PHE | PY | CHY | BAPY |
| Saponin | 0.5 | 30.4 ± 4.9 | 32.4 ± 3.2 | 20.3 ± 3.5 | 30.6 ± 5.3 |
|  | 1 | 49.7 ± 4.8 | 40.6 ± 4.2 | 38.8 ± 2.6 | 37.7 ± 3.7 |
|  | 2 | 41.2 ± 5.7 | 42.3 ± 4.3 | 40.4 ± 1.9 | 39.2 ± 2.5 |
|  | 5 | 46.8 ± 3.8 | 48.5 ± 6.2 | 36.2 ± 3.8 | 43.4 ± 1.6 |
|  | 10 | 34.3 ± 5.8 | 46.2 ± 2.3 | 30.8 ± 5.6 | 40.2 ± 3.5 |
| Rhamnolipid | 0.5 | 33.2 ± 2.1 | 40.2 ± 5.6 | 30.3 ± 2.4 | 27.4 ± 4.6 |
|  | 1 | 40.8 ± 5.8 | 43.6 ± 2.1 | 35.4 ± 7.1 | 39.8 ± 3 |
|  | 2 | 45.7 ± 7 | 40.9 ± 3.9 | 36.5 ± 4.3 | 38.9 ± 4.8 |
|  | 5 | 42.4 ± 1.3 | 44.2 ± 4 | 36.8 ± 5.5 | 41.4 ± 3.4 |
|  | 10 | 38.1 ± 3.7 | 40.6 ± 6.2 | 32.7 ± 6.1 | 33.1 ± 5.2 |
| Sophrolipid | 0.5 | 38.7 ± 4.8 | 47.6 ± 5.1 | 28.3 ± 4.6 | 27.3 ± 4.6 |
|  | 1 | 43.9 ± 5.1 | 44.9 ± 3.7 | 33.5 ± 3.6 | 32.4 ± 2.9 |
|  | 2 | 45 ± 2.4 | 58.4 ± 3.3 | 42.8 ± 2.9 | 31.3 ± 3.5 |
|  | 5 | 44.6 ± 1.5 | 53.2 ± 3.8 | 43.5 ± 3.8 | 28.7 ± 4.1 |
|  | 10 | 40.1 ± 5 | 53.7 ± 5.6 | 41.1 ± 5.8 | 22.1 ± 4.2 |
| Tween 20 | 0.5 | 21.4 ± 3 | 37.9 ± 2.1 | 20.4 ± 5 | 23.9 ± 5.3 |
|  | 1 | 38.3 ± 5.4 | 46.8 ± 3.7 | 33.2 ± 4.2 | 31.3 ± 2.7 |
|  | 2 | 40.7 ± 4.3 | 57.6 ± 1.8 | 43.6 ± 4.4 | 33.8 ± 3.8 |
|  | 5 | 42.5 ± 5.9 | 54.3 ± 4.9 | 46.7 ± 1.5 | 38.7 ± 5.1 |
|  | 10 | 38.2 ± 3.2 | 47.8 ± 2.8 | 32.5 ± 3.1 | 43.5 ± 4.2 |
